# Preparation and Photocatalytic Characterization of Modified Nano TiO_2_/Nd/Rice Husk Ash Material for Rifampicin Removal in Aqueous Solution

**DOI:** 10.1155/2022/2084906

**Published:** 2022-03-30

**Authors:** Thuy Dang Thi Ngoc, Ha Nguyen Thi, Dung Nguyen Duc, Sen Nguyen Thi, Toan Nguyen Duc, Nam Nguyen Hoang

**Affiliations:** ^1^Department of Environment, Hanoi University of Mining and Geology, Hanoi 100000/129000, Vietnam; ^2^Faculty of Environmental Sciences, VNU-University of Science, Vietnam National University, Hanoi 100000/11406, Vietnam; ^3^Institute of Natural Resources and Environment Science, 7th Floor, GIM Building, 460 Lane, Hanoi 100000/11408, Vietnam; ^4^Institute of Natural Resources and Environment Training, 83 Nguyen Chi Thanh, Hanoi 100000/11500, Vietnam

## Abstract

Antibiotics like rifampicin are often persistent in the environment. When entering the water, it causes antimicrobial resistance that affects the ecosystem and accumulates in the aquatic organisms and affects human health through the food chain. In this study, titanium dioxide was doped with neodymium (0.01 to 0.8%) using the sol-gel hydrothermal method. TiO_2_/Nd was then coated on rice husk ash to produce a modified TiO_2_/Nd/rice husk ash material containing 0.36% (w/w) Nd. The structural characteristics and photocatalytic properties of the materials were analyzed by X-ray diffraction, energy dispersive X-ray, transmission electron microscopy, scanning electron microscopy, forbidden zone energy, and specific surface area. The TiO_2_/Nd material exhibited a higher photocatalytic decomposition capacity than TiO_2_ and depended on the Nd content. The rifampicin removal efficiency of TiO_2_/Nd materials with 0.36 to 0.80% Nd contents was approximately 40% higher than that of TiO_2_/Nd containing 0.01 to 0.28% Nd. A new photocatalytic TiO_2_/Nd/rice husk ash material was developed to decompose rifampicin. The rifampicin-degrading efficiency of TiO_2_/Nd and TiO_2_/Nd/rice husk ash material reached approximately 86 and 75%, respectively, within 90 min under sunlight. Although a lower efficiency was obtained, the TiO_2_/Nd/rice husk ash material was selected to degrade rifampicin residue in water via the photocatalytic process (under sunlight) because of its advantages such as requirement of a small amount and easy recovery. In the rifampicin removal process, *k* values were found to match the zero- and first-order kinetics. In particular, for TiO_2_/Nd and TiO_2_/Nd/rice husk ash under solar irradiation, *R*^2^ values reached approximately 0.98. These results have been previously published as a preprint.

## 1. Introduction

Rifampicin is an antibiotic (molecular formula: C_43_H_58_N_4_O_12_) that is used in treating bacteria of the *Mycobacterium* strain, especially *M*. *tuberculosis* and *M*. *leprae*, and other *Mycobacterium* bacteria such as *M*. *bovis* and *M*. *avium*. The minimum inhibitory concentration observed for *M*. *tuberculosis* is 0.1–1.0 *μ*g/mL [1–3]. Antibiotic residues in water can cause adverse effects on aquatic organisms; particularly they develop antibiotic-resistant bacteria in the environment [4]. In addition, the presence of certain antibiotics in water, especially rifampicin, can significantly affect water treatment using biological methods [5].

The removal of antibiotic residues has been a topic of considerable interest to many scientists. Currently, several methods for removing antibiotic residues exist, such as absorption, advanced oxidation, biological treatment, and photocatalysis. Photocatalysis is one of the techniques that promise to not only degrade the persistent organic pollutants (POPs) that are difficult to decompose by biological methods and harmful microorganisms but also help remove certain antibiotics that are difficult or impossible to treat by biological methods. The characteristic of this type of catalysts is the production of pairs of electrons (e^−^) and holes (h^+^) under the effect of light, thereby creating compounds with strong oxidation properties and abundant electron supplies [6–9].

Nano TiO_2_ is widely used because of its photochemically stable catalytic properties, low cost, and nontoxicity to humans and the environment. Nano TiO_2_, which is used as a catalyst, is not influenced by reactions; therefore, it requires only initial investment for long-term use [10–12]. Therefore, nano-sized TiO_2_ has economic and technical advantages in inorganic-, organic-, and microbial-mediated removal processes [12]. However, because of the increasingly restricted zone energy of nano-sized TiO_2_ (3.05–3.25 eV), only the short-wavelength radiation (<380 nm) can activate the electrons from the valence to the conduction bands for photocatalytic activity [10, 13]. The ultraviolet rays of the solar radiation that reach the Earth's surface only account for approximately 4%, which limits the use of this natural source for the purpose of treating environmental problems with TiO_2_ [10, 14]. Therefore, it is necessary to reduce the band-gap energy of TiO_2_, which exhibits photocatalytic activity under visible light. Thus, several researchers have denatured TiO_2_ materials to reduce its bending energy (Eg) and extend the excitation light from the UV region to the visible area [10, 13, 14].

A number of recent surface modifications of TiO_2_ structures have been performed by introducing metal ions such as Zn, Fe, Cr, Eu, Y, Ag, and Ni and nonmetal ions such as N, C, S, F, and Cl. These materials have been shown to be effective in enhancing the photocatalytic activity in the visible light [10, 14–19].

In addition, the recovery of small and dispersed TiO_2_ nanoparticles is difficult, thereby reducing its reusability. Therefore, to reduce the product cost, it is necessary to attach TiO_2_ nanoparticles to a carrier with a large surface area. These carriers should have the following characteristics: good bonding to the catalyst, no catalytic decomposition effect, a large surface area, and an affinity for adsorption with pollutants. Typically used carriers include beeswax, activated carbon, glass, silica gel, polymer materials, zeolite, cotton, and cellulose.

Husk cover and rice husk ash are agricultural wastes, accounting for approximately one-fifth of the world's annual production of rice (approximately 545 million tons per annum). Globally, more than 20 million tons of husk ash is released annually [20]. The amount of ash that enters the ecosystem can be damaging to humans and animals and may result in silicosis, respiratory failure, and death. Husk ash is a good adsorbent and can be used for treating many inorganic and organic pollutants [21–23]. Thus, a portion of the agricultural waste is utilized, thereby reducing the amount of waste rice husk [21]. In addition, husk ash is used as a carrier for catalytic materials because of its good mechanical stability and chemical inertness; when used as a carrier, it facilitates the separation of the catalyst from the solution after the reaction. Furthermore, this material absorbs nitrogen compounds, such as rifampicin [24].

Nitrogen-containing organic compounds such as rifampicin were adsorbed onto rice husk ash coupled with the TiO_2_ nanoparticle, which acted as a photochemical catalyst, converting rifampicin to CO_2_, H_2_O, and N_2_ (nontoxic). Therefore, a new photocatalytic TiO_2_/Nd/rice husk ash material was developed to decompose rifampicin. This approach minimizes environmental pollution from agricultural waste, increases the efficiency of nanomaterials, saves material-related cost, and does not necessitate postprocessing treatments. This study provides a new approach of nitrogen compound treatment or removal and can be applied in the industries [25].

## 2. Materials and Methods

### 2.1. Chemicals and Equipment

**Figure 1:**
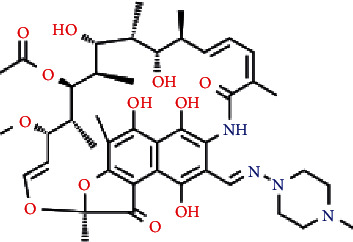
The chemical structure of rifampicin.

The chemicals used in this study included TiCl_4_, NH_4_NO_3_, (NH_2_)_2_CO, rifampicin (see Figure 1), methanol, H_2_SO_4_, HNO_3_, Na_2_CO_3_, NaOH, NaH_2_PO_4_, Na_2_HPO_4_, NdCl_3_, and PVA. The chemical compounds have a purity of pure for analysis (PA), produced by Merck, Germany, distilled water, and ultra-distilled water.

Husk was obtained from the rice husk enterprise in Hoai Duc, Ha Noi. The rice husks were washed with distilled water to remove impurities, dried at 105°C for 2 h, and then placed in a furnace maintained at a temperature of 800°C for 3 h in a N_2_ gas environment to obtain ash-bearing material. The product was ground to a size of approximately 0.1–0.5 mm.

Rifampicin solution was prepared with a concentration of 20 mg/L. Then, 0.5 M TiCl_4_ was prepared by diluting 3 M TiCl_4_ with cold water.

Phosphate buffer solution (pH 7.4) was prepared using NaH_2_PO_4_ and Na_2_HPO_4_ [26].

Instruments used to perform the experiments consisted of a drying cabinet produced by Germany, an oven (Carbolite model AAF-11/7, England), and a heating magnetic stirrer (IKA, Germany).

### 2.2. Preparation of Modified Nano TiO_2_/Nd Material Coated in Husk Ash by the Sol-Gel Hydrothermal Method

#### 2.2.1. Preparation of Modified Nano TiO_2_/Nd Material by the Sol-Gel Hydrothermal Method

Nd-modified nano TiO_2_ material was prepared by the sol-gel hydrothermal method according to the method described by Nam et al. [27]. The composition and ratio of used chemicals are shown in Table 1. First, 60 mL of 1 M NH_4_NO_3_ was mixed with 450 mL of 1 M (NH_2_)_2_CO, 180 mL of 1 M PVA, and 60 mL of 0.5 M TiCl_4_ with 1 g/L Nd ranging from 0.1 to 1.0 mL. The mixture was heated to 70°C and stirred continuously for 24 h at 1200 rpm and then heated up to 90°C and stirred for 12 h. The sol-gel solution obtained was dried at 120°C for 12 h, following which the temperature was increased to 250°C for 3 h. The mixture was heated until the release of white smoke ceased, and a black powder was generated. Finally, the materials were placed in a furnace at 600°C for 3 h and heated at a rate of 10°C/min. Thereafter, the furnace was washed 4-5 times with distilled water and superclean water and then dried at 120°C for 2 h [27].

#### 2.2.2. Coating Nano TiO_2_/Nd Material on Rice Husk Ash

Husk ash was added to the prepared sol-gel solution such that the concentrations of TiO_2_/Nd in husk ash corresponded to 0.1, 0.2, 0.3, 0.4, and 0.5% w/w. Thermal modification process was initiated by stirring the mixture at 90°C for 12 h. This was followed by similar processes as in the preparation of Nd-modified TiO_2_ nanomaterials by the sol-gel hydrothermal method.

### 2.3. Rifampicin Removal by Nano TiO_2_/Nd and Nano TiO_2_/Nd/Husk Ash Materials

For rifampicin removal, 0.1 g of powdered nano TiO_2_/Nd material or 1.0 g nano TiO_2_/Nd material/husk ash was transferred to 200 mL glass cups containing 100 mL rifampicin solution (20 mg/L) and stirred at 100 rpm. The experiments were conducted under natural light conditions (day and night). Natural light has an illumination intensity of 20.000 Lux. Samples were withdrawn at 0, 15, 30, 45, 60, 75, and 90 min and analyzed for the residual rifampicin concentration.

### 2.4. Analysis Methods

The surface area of the husk ash was determined by the BET analysis in a N_2_ environment at 196°C using a NOVA 1200 Quanta chrome (USA). The surface structures of the husk ash and TiO_2_ material/husk ash were characterized using scanning electron microscopy (SEM) analysis (JEOL 5410 LV, Japan). The purity of the TiO_2_ material was determined using X-ray diffraction (XRD) (Siemens D5005, Germany). TiO_2_ nanoparticle size was determined by transmission electron microscopy (TEM) (LIBRA120, Germany). The elemental compositions were analyzed by energy dispersive X-ray (EDX) analysis (JEOL 6490JED 2300, 2300, Japan).

Rifampicin concentration was analyzed using the molecular absorption spectroscopic method as described by Benetton et al. [28]. A calibration curve was prepared using UV-VIS Optizen 2120UV (England).

### 2.5. Rifampicin Degradation Kinetic Calculations

The degradation rate of rifampicin and the reaction order presenting the best fit were investigated by plotting the residual rifampicin concentration versus time, which was analyzed using zero-order, first-order, and second-order kinetic models [29]:

Zero-order reaction:
  (1) $$\begin{array}{c} C=\;C_{0}-kt. \end{array}$$
First-order reaction:
  (2) $$\begin{array}{c} C=\;\ln\;C_{0}-kt⟶C=\;C_{0}e^{-kt}. \end{array}$$
Second-order reaction:
  (3) $$\begin{array}{c} \frac{1}{C}=\frac{1}{C_{0}}+kt\frac{1}{C}, \end{array}$$
where *C*_*0*_ is the concentration of the reactants at time zero, *C* is the concentration after reaction time *t*, and *k* is the reaction rate constant.
Based on the obtained kinetic graphs, the regression coefficients and kinetic parameters, such as the apparent order degradation rate constant (*k*), were identified.

## 3. Results and Discussion

### 3.1. Characteristics of Modified TiO_2_/Nd Powdered Material

Synthesized TiO_2_/Nd material samples using different Nd concentrations were analyzed for their structure, photolysis properties, and surface properties using EDX, XRD, and SEM analysis.

#### 3.1.1. Morphology of Nano TiO_2_/Nd-Modified Material

**Figure 2:**
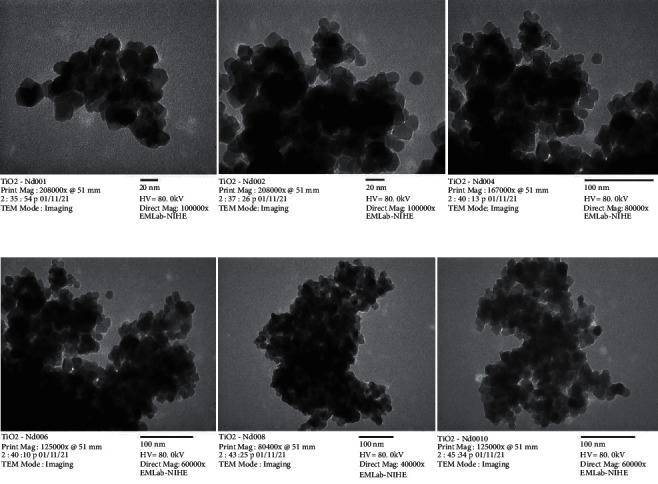
TEM image of TiO_2_/Nd-modified nanomaterials with different Nd contents.

The size and the crystal forms of the prepared material were analyzed by conducting particle size analysis using TEM and XRD. The morphology of the modified nano TiO_2_/Nd materials using the sol-gel hydrothermal method is shown in Figure 2.

At different Nd concentrations, the materials in the anatase form are even and have a relatively even particle size of approximately <20 nm. Compared with the results of the nanomaterials synthesized by the sol-gel method [27], the nano TiO_2_/Nd-modified materials exhibit particle sizes that are substantially smaller than those of the nonmodified materials. This result is consistent with the findings of the study by Huang et al. [30] and Nam et al. [27].

#### 3.1.2. The XRD Spectrum of the Nano TiO_2_/Nd-Modified Materials

**Figure 3:**
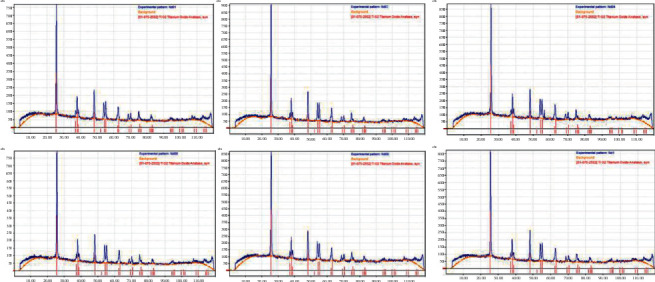
XRD images of nano TiO_2_/Nd-modified materials with different Nd contents.

X-ray diffraction (XRD) patterns of the nonmodified nano TiO_2_ materials and the modified nano TiO_2_/Nd materials are shown in Figure 3. All peaks are sharp, and no strange diffraction peaks appear, proving that the prepared TiO_2_ materials have a characteristic crystal structure. Characteristic diffraction peaks at 2*θ* values are approximately 25.36 (101), 37.93 (004), 48.07 (200), 5 4.03 (105), 55.13 (211), and 62.81 (204), indicating that the nanomaterial obtained exists only in the anatase phase. In addition, no characteristic peaks corresponding to the rutile and brookite phases were observed. Lattice parameters, particle size, and density of nano TiO_2_/Nd-modified materials with different Nd contents are presented in Table 2.

The results showed the insertion of Nd^3+^ ions into the crystal structure of the TiO_2_ materials. Although prepared with different Nd contents, the nanomaterials obtained are pure, single-phase, without the appearance of diffraction peaks of rare earth ions. In addition, the crystal lattice parameters of materials with different Nd contents observed variations in the lattice constants *a* and *c*, which was found to be larger in the modified nano TiO_2_/Nd material than that of the nonmodified materials. This can be explained by the fact that the radius of the Nd^3+^ ion is larger than that of the Ti^4+^ ion. When the Nd^3+^ ion replaces the position of the Ti^4+^ ion in the crystal lattice, it increases the crystal size [31, 32].

However, the size of the crystal lattice and the density of all six materials were practically identical. In addition, these findings are similar to those of some previous studies where the cell lattice size of the material prepared by the sol-gel hydrothermal method was smaller with larger density when modified with metals [30–32].

#### 3.1.3. EDX Spectrum of Nano TiO_2_/Nd Materials

**Figure 4:**
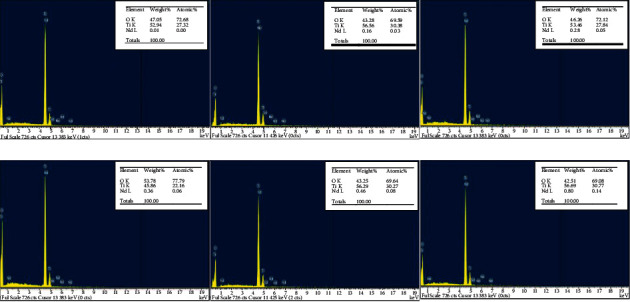
EDX images of nano TiO_2_/Nd-modified materials with different Nd contents.

The percentages of elements contained in the nano TiO_2_/Nd materials with different Nd contents are shown in Figure 4.

The peaks in the EDX diagrams indicate the presence of Ti, O, and Nd in the material samples. Peaks of other elements were not observed. This proves that the synthetic nano TiO_2_ materials were highly pure. The results of the quantitative analysis of the material compositions showed that the Ti content accounted for between 46 and 57% by weight and from 27 to 31% by atomic numbers, and the O content was found in the range of 42–54% and 69–78% by weight and atomic numbers, respectively. In addition to the Ti and O peaks, Nd peaks were observed with a relatively small intensity, accounting for only 0.01–0.8% and 0.01–0.14% by weight and atomic number, respectively.

Anatase nanomaterials were evenly arranged in a single-crystal form. In the crystal lattice of TiO_2_, the Ti atoms in some vertices were replaced by Nd atoms. However, because Ti has a valency of IV, it forms a bond with the surrounding four O atoms. Nd has a valency of III; thus, when the position of the Ti atom is replaced by Nd, the Nd atom creates an electric imbalance. In addition, OH^−^ groups are produced on the surface of TiO_2_ by the water separation process of oxides. These groups can exist in free or surface-bonded states via hydrogen bonds, which form several molecular layers on the TiO_2_ surface. The OH^−^ group can trap holes and absorb water molecules, which provides electrons to form reduced hydroxyl radicals. Therefore, the recombination rate is reduced between electron and hole pairs, which indicates that the photocatalytic activity of TiO_2_ is enhanced when modified by the Nd^3+^ ion [31].

#### 3.1.4. Eg Values of Nano TiO_2_/Nd-Modified Material

**Figure 5:**
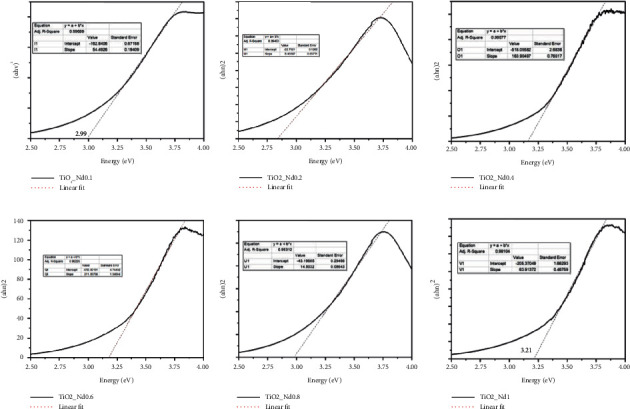
Eg spectrum of nano TiO_2_/Nd-modified materials with different Nd contents.

As shown in Figure 5, the lowest Eg value was observed for the Nd content of 0.16%, while the highest Eg value was observed for the material with an Nd content of 0.8%, reaching 3.21 eV. Thus, the forbidden zone energy of the Nd-modified material is generally lower than that of the nonmodified one, which considerably improves the ability of these modified materials to absorb the visible light compared with that of nano TiO_2_ material. For nonmodified TiO_2_, the maximum energy absorption occurs in the ultraviolet radiation region (*λ* < 400 nm) with electron excitation from the 2p orbital of the O atom to the 3d orbital of Ti. Therefore, the Eg value of TiO_2_/Nd materials with different Nd contents significantly decreases to below 2.89 eV. Thus, visible light can excite electrons from the intermediate energy level (4f) to the conductive energy region. This is also consistent with studies on TiO_2_ modified using rare earth elements [33, 34].

#### 3.1.5. Nano TiO_2_/Nd Materials Coated on Rice Husk Ash

**Figure 6:**
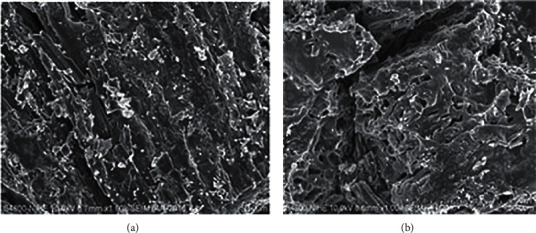
SEM images of rice husk ash (a) and nano TiO_2_/Nd/rice husk ash (b).

The modified nano TiO_2_/Nd materials coated on rice husk ash were prepared from a sol-gel solution similar to those used to make modified nano TiO_2_/Nd materials with an Nd content of 0.36%. Their photocatalytic activity was compared to TiO_2_ nanoparticles. The SEM image of the nano TiO_2_/Nd/rice husk ash material shows that TiO_2_/Nd nanoparticles are attached at the crevices and on the holes of the material surface and are fairly evenly distributed throughout the surface of the rice husk ash. The results also indicated that the coatings on the rice husk ash were homogeneous (see Figure 6).

**Figure 7:**
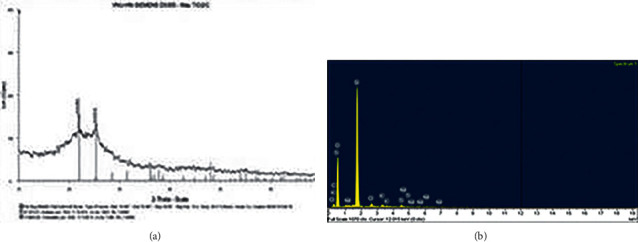
XRD (a) and EDX (b) spectra of nano TiO_2_/Nd/rice husk ash.

Figure 7(a) shows the characterized peaks at 2*θ* = 25.3° for TiO_2_ and 22° for SiO_2_, indicating the presence of TiO_2_ and SiO_2_ particles on the surface of the TiO_2_/Nd/rice husk ash material. The mass and percentage of elements found in the TiO_2_/Nd/rice husk ash material are presented in Figure 6(b). The findings show that the material consists mainly of Si, accounting for 83.13%, and O and C, accounting for 6.33% and 5.64%, respectively. However, Ti only occupies a small amount (0.19% *w*/*w*), whereas a small amount of other elements such as Nd, K, Cl, and Ca were found to be derived from rice husk ash. This shows that the nano TiO_2_/Nd material was successfully coated on rice husk ash by the sol-gel hydrothermal method because of its high absorption capacity and photocatalytic activity. Using this material allows continuous operation of the treatment process without separating the material after treatment. Therefore, it increases the removal efficiency and also reduces the treatment cost.

### 3.2. Removal Efficiency of Nano Nd-TiO_2_ and Nano Nd-TiO_2_/Rice Husk Ash Materials for Rifampicin

**Figure 8:**
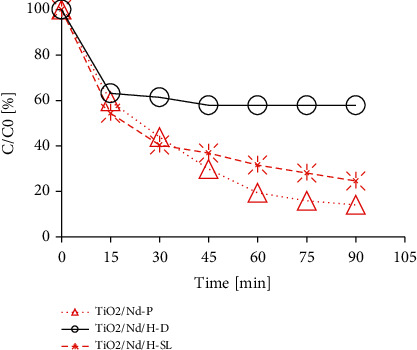
Rifampicin removal efficiency of nano TiO_2_/Nd and TiO_2_/Nd/rice husk ash under different conditions (TiO_2_/Nd-P: nano TiO_2_/Nd, natural light; TiO_2_/Nd/H-SL:TiO_2_/Nd/rice husk ash, natural light; TiO_2_/Nd/H-D: TiO_2_/Nd/rice husk ash, dark).

The results of the photocatalytic decomposition of rifampicin antibiotic in the presence of TiO_2_/Nd and nano TiO_2_/Nd/rice husk ash materials are shown in Figure 8. The results clearly show that both types of materials exhibit photocatalytic activity. However, the activity of TiO_2_/Nd/rice husk ash material is lower (approximately 10% after 90 min) compared with that of nano TiO_2_/Nd material. This difference is mainly related to significant differences in the content of nano TiO_2_, the light absorption ability, and the rifampicin's interaction with the rice husk ash material. Although both materials are prepared from same-size nanoparticles and have the same Eg, the BET surface areas of TiO_2_/Nd and nano TiO_2_/Nd/rice husk ash materials are 58.97 and 107 m^2^/g, respectively. The size of the TiO_2_/Nd/rice husk ash was considerably larger than that of the TiO_2_/Nd nanoparticle. Because of the bulky molecular structure of rifampicin with many functional groups such as --OH, >NH, and >C=O, it can easily bond with the metals of the adsorbents. This is more evident in the experimental results under dark conditions (see Figure 8). In addition, the rapid absorption rate shows that the maximum absorbability is achieved after 15 min.

Under natural light conditions, the photocatalytic efficiencies of both materials markedly increased over reaction time; however, rifampicin degradation efficiency by TiO_2_/Nd nanoparticles was higher. This is related to the absorption process and the size of the rifampicin molecule. Therefore, the photocatalytic process occurs simultaneously during absorption, as shown in the following reactions [35]:

(4) $$\begin{array}{c} {\text{TiO}}_{2}+\text{ hv}⟶{\text{TiO}}_{2}\left({\text{h}}^{+}{+\text{e}}^{-}\right),\\ {\text{TiO}}_{2}\left({\text{h}}^{+}\right){+\text{H}}_{2}\text{O}{⟶}^{*}{\text{OH}+\text{H}}^{+}{+\text{TiO}}_{2},\\ {\text{TiO}}_{2}\left({\text{h}}^{+}\right){+\text{OH}}^{-}{⟶}^{*}{\text{OH}+\text{TiO}}_{2},\\ {\text{TiO}}_{2}\left({\text{h}}^{+}\right){+\text{O}}_{2}{⟶}^{*}{\text{O}}_{2}{+\text{TiO}}_{2}. \end{array}$$

The activated molecules react with the molecules of rifampicin to produce CO_2_ and H_2_O.

(5) $$\begin{array}{c} {\text{C}}_{43}{\text{H}}_{58}{\text{N}}_{4}{\text{O}}_{12}+\frac{103}{2}\left({}^{*}{\text{O}}_{2}\right)⟶{43\text{CO}}_{2}{+29\text{H}}_{2}{\text{O}+2\text{N}}_{2},\\ {\text{C}}_{43}{\text{H}}_{58}{\text{N}}_{4}{\text{O}}_{12}+194\left({}^{*}\text{OH}\right)⟶{43\text{CO}}_{2}{+66\text{H}}_{2}{\text{O}+2\text{N}}_{2}. \end{array}$$

From the reaction equations, a large number of activated molecules are required to oxidize C_43_H_58_N_4_O_12_, indicating that a large amount of H^+^ is needed for the reaction.

At the early stage, when rifampicin molecules are rapidly adsorbed to the surface of the material, OH^−^ ions and O_2_ molecules interact with the material surface, thereby reducing the formation of activated molecules and facilitating the recombination of electrons and holes. Therefore, the rifampicin removal efficiency via photochemical reactions was insignificant. As the reaction time extended, the rates of photochemical reactions of both materials markedly increased. The concentration of rifampicin tends to significantly decrease for nano TiO_2_/Nd materials when compared with that of TiO_2_/Nd/rice husk ash. This is probably because the rice husk ash accounts for a large proportion of the TiO_2_/Nd/rice husk ash material (see SEM and EDX in Figures 6 and 7).

In the TiO_2_/Nd/rice husk ash material, TiO_2_/Nd nanoparticles occupy only a small portion of the surface area of the material; therefore, when rifampicin molecules are absorbed onto the rice husk ash material, the interaction between the activated molecules and rifampicin has a lower probability than that of the nano TiO_2_/Nd material. In contrast, because of the larger size of the TiO_2_/Nd/rice husk ash material, it prevents light from passing deep into the solution, which decreases the decomposition efficiency and increases the reaction time. Rifampicin decomposition efficiency of the TiO_2_/Nd/rice husk ash material reached >75% when the reaction time reached up to 90 min.

### 3.3. Rifampicin Degradation Kinetics

**Figure 9:**
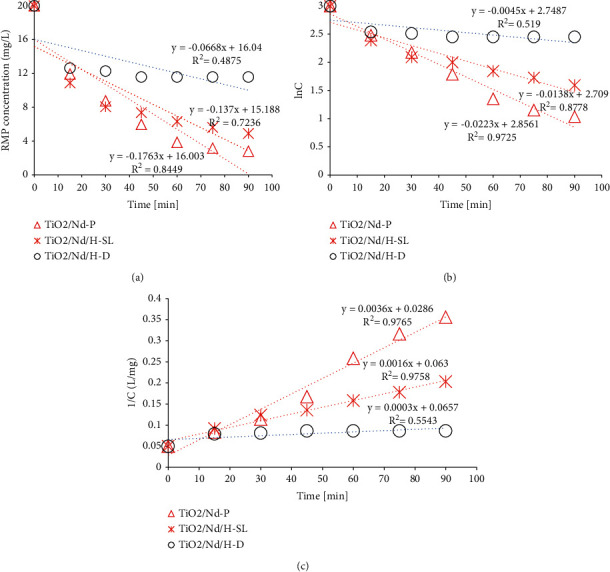
Concentration of rifampicin remaining versus time according to (a) zero-order reaction, (b) first-order reaction, and (c) second-order reaction.

The results of the kinetic calculations for rifampicin removal according to the zero-, first-, and second-order equations are shown in Figure 9.

The summarized results in Table 3 show that for all three kinetics, the TiO_2_/Nd powder presented the highest *k* value and regression correlation, *R*^2^. The *k* values of the zero- and first-order kinetics are similar to the maximum and minimum *k* values, respectively, which were reported in the study by Cizmic et al. [36].

The Langmuir–Hinshelwood kinetics are applied with first-order kinetic similarities, which considers simultaneously occurring absorption processes and the decomposition reaction. Compared with the powdered TiO_2_/Nd material, the TiO_2_/Nd material coated with rice husk ash under natural light conditions (-SL) displayed lower *k* and *R*^2^ values; however, these were observed to be better than those of TiO_2_/Nd/H under dark irradiation. In a study by Vaucher et al., the kinetics of telithromycin photodegradation and oxidative degradation were determined, where both degradation approaches followed first-order reaction kinetics [29].

The pseudo-first-order kinetic model has been commonly used in numerous studies to investigate the antibiotic degradation process under different conditions [36–38], and photocatalytic degradation of sulfamethazine in aqueous solution using ZnO has been reported. The findings showed that 78 and 95% of sulfamethazine were degraded after 60 min of irradiation without and with ZnO, respectively, and the rate constant *k* obtained was 2.58 × 10^−2^ and 4.95 × 10^−2^ min^−1^, respectively.

The Langmuir–Hinshelwood model was studied by Kais for the photocatalytic degradation of rifampicin and tetracycline [39]. The influence of certain parameters and the kinetic model was investigated. The results showed that the apparent rate constant (*k*_app_) and initial rate constant (*r*_0_) decreased during an increase in the initial rifampicin concentration. In one study, the amount of photocatalyst (TiO_2_) and pH were considered to be significant factors affecting the antibiotic degradation process [40, 41].

## 4. Conclusions

In this study, anatase nano TiO_2_ materials were modified with different Nd contents by the sol-gel hydrothermal method using a TiCl_4_ solution at a temperature of 600°C. The material properties and photocatalytic reactions of the nano TiO_2_/Nd and TiO_2_/Nd/rice husk ash materials were investigated using modern techniques. The results showed that Nd doping changed the width of the forbidden zone, thereby affecting the electronic transition energy. The modification notably enhanced the visible light photocatalytic activity of modified TiO_2_ compared with that of pure TiO_2_ and depended on the Nd content. The rifampicin decomposition efficiency of nano TiO_2_ material with 0.36% Nd under natural light reached approximately 86% after 90 min. The TiO_2_/Nd/rice husk ash material was successfully prepared when TiO_2_/Nd was coated on rice husk ash by the sol-gel hydrothermal method, with a Ti content of approximately 0.19% (*w*/*w*). Although its photocatalytic reaction efficiency for rifampicin decomposition was lower than that of nano Nd-TiO_2_ materials (more than 75% after 90 min), this material is suggested because after the photocatalytic process, the Nd-TiO_2_ material is still retained on the rice husk ash carrier and are not released into the solution and do not cause secondary environmental pollution. The Nd-TiO_2_/rice husk ash material can be recovered by a simple method and can be used for a long time.

The kinetics of rifampicin removal followed the zero- and first-order reaction kinetics, especially for Nd-TiO_2_ material in powder form (TiO_2_/Nd-P) and Nd-TiO_2_/rice husk ash material under sun light (TiO_2_/Nd/H-SL). The *k* and *R*^2^ values of TiO_2_/Nd/H-D were similar and remarkably lower than those of TiO_2_/Nd and TiO_2_/Nd/H under solar light.

## Figures and Tables

**Table 1:** Composition and ratio of chemicals in prepared nano TiO_2_-modified Nd by the sol-gel hydrothermal method.

Chemical	NH_4_NO_3_ (1.0 M)	(NH_2_)_2_CO (1.0 M)	PVA (1.0 M)	TiCl_4_ (0.5 M)	Nd^3+^ (1.0 g/L)
Volume (mL)	60	450	180	60	0.1–1.0

**Table 2:** Lattice parameters, particle size, and density of nano TiO_2_/Nd-modified materials with different Nd contents.

Nd/TiO_2_ ratio (% *w*/*w*)	Samples	Lattice parameters		Density, *D* (g/cm^3^)
		*a* = *b* (Å)	*c* (Å)	
0.01	TiO_2_-Nd 01	3.770	9.420	3.962
0.16	TiO_2_-Nd 02			
0.28	TiO_2_-Nd 04			
0.36	TiO_2_-Nd 06			
0.46	TiO_2_-Nd 08			
0.80	TiO_2_-Nd 1			

**Table 3:** Kinetics calculations: reaction rate constant and regression coefficients.

Materials	Zero-order kinetic		First-order kinetic		Second-order kinetic	
	*k*	*R* ^2^	*k*	*R* ^2^	*k*	*R* ^2^
TiO_2_/Nd-P	0.1763	0.85	0.0223	0.97	0.0036	0.98
TiO_2_/Nd-SL	0.137	0.72	0.0138	0.88	0.0016	0.98
TiO_2_/Nd/H-D	0.0668	0.49	0.0045	0.52	0.0004	0.66

## Data Availability

(1) The data are all carried out at our laboratories at the Department of Environment, Hanoi University of Mining and Geology, Faculty of Environmental Sciences, VNU University of Science and other collaborated partners. (2) The data in the manuscript can be accessed at the Department of Environment, Hanoi University of Mining and Geology, Faculty of Environmental Sciences, VNU University of Science and other collaborated partners.
